# Interactive effects of plant neighbourhood and ontogeny on insect herbivory and plant defensive traits

**DOI:** 10.1038/s41598-017-04314-3

**Published:** 2017-06-22

**Authors:** Xoaquín Moreira, Gaétan Glauser, Luis Abdala-Roberts

**Affiliations:** 1 Misión Biológica de Galicia (MBG-CSIC), Apartado de correos 28, Pontevedra, Galicia 36080 Spain; 2 Neuchâtel Platform of Analytical Chemistry, University of Neuchâtel, Rue Emile Argand 11, Neuchâtel, 2000 Switzerland; 3 Departamento de Ecología Tropical, Campus de Ciencias Biológicas y Agropecuarias, Universidad Autόnoma de Yucatán, Apartado Postal 4-116, Itzimná, Mérida, Yucatán 97000 Mexico

## Abstract

Plant ontogenetic stage and features of surrounding plant neighbourhoods can strongly influence herbivory and defences on focal plants. However, the effects of both factors have been assessed independently in previous studies. Here we tested for the independent and interactive effects of neighbourhood type (low vs. high frequency of our focal plant species in heterospecific stands) and ontogeny on leaf herbivory, physical traits and chemical defences of the English oak *Quercus robur*. We further tested whether plant traits were associated with neighbourhood and ontogenetic effects on herbivory. We found that leaf herbivory decreased in stands with a low frequency of *Q. robur*, and that saplings received less herbivory than adult trees. Interestingly, we also found interactive effects of these factors where a difference in damage between saplings and adult trees was only observed in stands with a high frequency of *Q. robur*. We also found strong ontogenetic differences in leaf traits where saplings had more defended leaves than adult trees, and this difference in turn explained ontogenetic differences in herbivory. Plant trait variation did not explain the neighbourhood effect on herbivory. This study builds towards a better understanding of the concurrent effects of plant individual- and community-level characteristics influencing plant-herbivore interactions.

## Introduction

Plants grow in heterogeneous environments where the presence of conspecific or heterospecific neighbouring plants can greatly affect their growth, survival and reproduction^1, 2^, as well as their interactions with antagonists^3–5^ and mutualists^6^. For example, in the case of plant-herbivore interactions, the frequency of a specific host plant species is lower in heterospecific relative to conspecific neighbourhoods (assuming total plant density is held constant in both cases) and this reduces the chance that herbivores will find and feed on focal plants (Resource Concentration Hypothesis^7^). Similarly, herbivore attack on host plant species may decrease in heterospecific relative to conspecific neighbourhoods due to the presence of one or more plant species which attract herbivores or interfere with herbivore location of the preferred host plant (i.e., associational resistance^8^). These differences in patterns of herbivore attack are in turn expected to drive concomitant changes in plant defence investment in response to damage^8^. Alternatively, studies have also reported that plant neighbourhood diversity or species composition can indirectly affect herbivory on focal plants by modifying plant nutritional quality (e.g., physical traits and secondary metabolites^9–12^), independently of resource concentration or associational effects. For example, competition for resources or facilitation among heterospecific plants or changes in abiotic conditions may alter plant growth or the nutritional value of plant tissues to herbivores^13–15^. However, despite a rich body of work on the effects of plant neighbourhood features (e.g., plant density, focal species frequency, species composition) on plant-herbivore interactions^8, 16, 17^, findings are highly variable among studies and the mechanisms underlying observed patterns in many cases remain poorly understood^8, 18^.

Plant investment in defences is known to vary with ontogeny^19^. In woody species, defence investment is expected to increase from the sapling to the young adult (pre-reproductive) stage once resource reserves are accumulated and the plant can increase relative allocation to defences^19, 20^. Subsequently, defence investment may be maintained or decrease during the adult stage^19, 20^. In addition to endogenous processes such as allocation constraints, plant variation in defence investment may also be explained by the risk of being attacked by herbivores. According to classic theory, plants that are easier to find (i.e., more apparent) either because they are larger in size, more abundant, or long-lived should invest more in defences than smaller, less abundant or ephemeral plants (Plant Apparency Theory^21^). Although studies testing this prediction have mostly focused on inter-specific comparisons, we could extend this framework to make predictions about intra-specific variation, particularly that occurring over ontogeny. In this case, adult plants, which are visually (due to their larger size) or chemically (due to greater emission of volatiles used as cues by herbivores) more conspicuous than young plants, should be easier to detect by insect herbivores and therefore experience higher herbivory and in turn greater investment in defences.

To date, theory on neighbourhood effects and the ontogeny of plant defence have developed independently for the most part, despite there being important insights to be gained from merging the two. The importance of joining these bodies of research resides in that there is a large potential for neighbourhood features and ontogenetic variation to interactively shape plant defences and herbivory. Neighbourhood effects on herbivory may be contingent on plant ontogeny if young and adult individuals vary in the risk of being detected by herbivores or if neighbourhood effects on plant defences (via plant-plant interactions) vary with plant age (e.g., due to differences in allocation constraints) and this in turn influences damage. Likewise, predictions for ontogenetic differences in herbivory and allocation to defences may vary depending on the neighbourhood context, where for example differences in apparency between adult and young plants of a focal plant species might be weaker in heterospecific relative to conspecific neighbourhoods, o﻿r similarly, ﻿﻿﻿in heterospecific neighbourhoods with﻿ a high vs. low frequency of ﻿that﻿ host plant (i.e., resource concentration effects increasingly overrule ontogenetic differences with decreasing host plant frequency).

In the present study, we tested for the independent and interactive effects of plant neighbourhood type (low vs. high frequency of a focal plant species in heterospecific stands) and ontogenetic stage on insect leaf herbivory, and leaf physical traits (water content and specific leaf area), and leaf chemical defences (phenolic compounds) in the English oak, *Quercus robur* L. (Fagaceae), a long-lived tree common throughout western Europe. To understand the linkage between plant traits and herbivory, we further tested whether and which plant traits were associated (and potentially mediated) neighbourhood and ontogenetic effects on *Q. robur* leaf herbivory. To this end, we surveyed saplings and adult trees of *Q. robur* found in 20 field sites across north-west Spain. At each site, we selected two adjacent stands similar in size and total plant density: one with a high frequency of *Q. robur* individuals (>85% of the adult individuals were of the focal species) and another with a low frequency of this species (<35% of the adult individuals were of the focal species). In both cases, stands were composed of *Q. robur* plus two other tree species; the identity of these non-focal species was the same in all cases (i.e., tree species composition was held constant across stands). In addressing the above, this study builds towards a better understanding of the combined effects of plant neighbourhood and ontogeny on plant-herbivore interactions and plant traits associated with such effects.

## Results

### Effects of neighbourhood type and ontogeny on *Q. robur* leaf herbivory and leaf traits

#### Leaf herbivory

**Figure 1:**
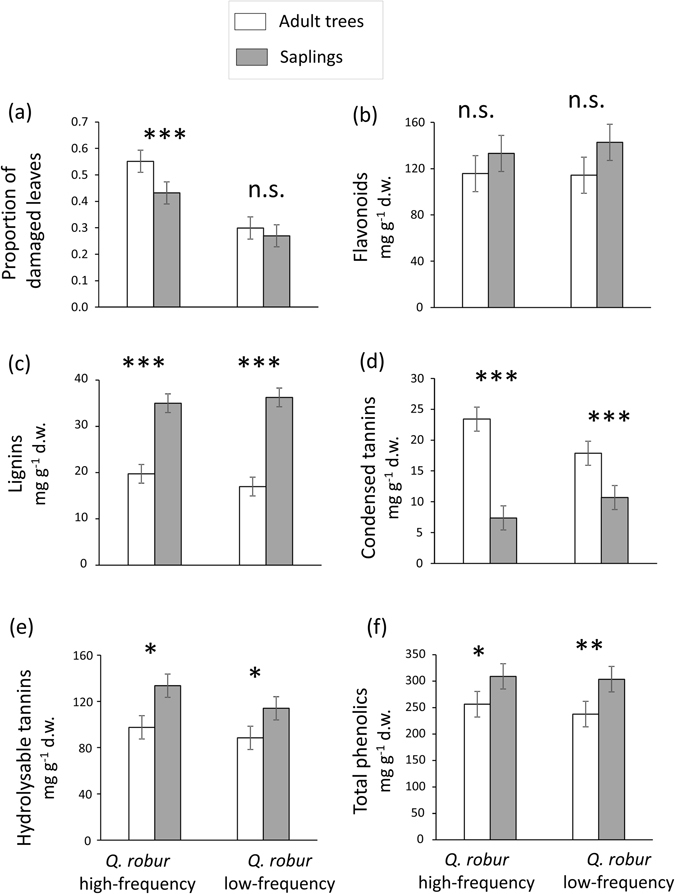
Effect of plant neighbourhood composition and ontogeny on herbivory and plant chemical defences in *Quercus﻿ robur*. (**a**) Proportion of leaves damaged by insect herbivores, and concentration (mg g^−1^ d.w.) of leaf (**b**) flavonoids, (**c**) lignins, (**d**) condensed tannins, (**e**) hydrolysable tannins, and (**f**) total phenolics for adult reproductive trees (white bars) and saplings (grey bars) growing in *Q. robur* high-frequency and low-frequency stands. Bars are least square means ± standard error (N = 60). Asterisks indicate significant ontogenetic differences within each *Q. robur* stand type at *P* < 0.05 (*), *P* < 0.01 (**) and *P* < 0.001 (***). n.s. = non-significant.

Both neighbourhood type and ontogenetic stage significantly affected leaf herbivory in *Q. robur* (Table 1, Fig. 1a). Leaf herbivory on individuals growing in *Q. robur* low-frequency stands was 42% lower than on those growing in high-frequency stands (Fig. 1a), and herbivory was 17% lower on saplings than on adult trees (Fig. 1a). However, beyond these independent effects we also found a significant interaction term where the difference in herbivory between saplings and adult trees was significant in *Q. robur* high-frequency stands but not in low-frequency stands (Fig. 1a), and where the magnitude of reduction in herbivory in low- vs. high-frequency stands was greater for adults (46%) compared with saplings (37%) (Fig. 1a).

**Table 1:** Summary of results from mixed models testing for the effects of neighbourhood type (high- vs low-frequency of *Quercus robur*), ontogenetic stage (adult trees vs. saplings) and their interaction on leaf herbivory (proportion of herbivore-damaged leaves), leaf physical traits (proportion of water content and specific leaf area [SLA]) and concentration of leaf chemical defences (flavonoids, lignins, condensed and hydrolysable tannins and total phenolics) in *Q. robur*.

	Frequency		Ontogeny		Frequency × ontogeny	
	F_1,19_	*P*-value	F_1,198_	*P*-value	F_1,198_	*P*-value
Leaf herbivory	20.24	<**0.001**	11.45	<**0.001**	4.32	**0.039**
Flavonoids	0.31	0.581	0.25	0.616	0.76	0.384
Lignins	0.17	0.685	116.36	<**0.001**	1.56	0.213
Condensed tannins	0.27	0.608	96.80	<**0.001**	4.55	**0.034**
Hydrolysable tannins	3.48	0.078	8.45	**0.004**	0.00	0.996
Total phenolics	0.86	0.365	11.85	**0.001**	0.49	0.483
Water content	0.44	0.514	0.56	0.454	3.78	0.053
SLA	0.93	0.346	118.13	<**0.001**	0.76	0.384

Herbivory was estimated as the proportion of leaves damaged by insect herbivores for two randomly chosen low-hanging branches. Herbivory data were logit-transformed to achieve normality of residuals. Site and site × neighbourhood type were included as random factors. F-values, degrees of freedom and associated *P*-values of fixed factors are reported. Significant *P*-values (*P* < 0.05) are typed in bold.

#### Chemical defences

**Figure 2:**
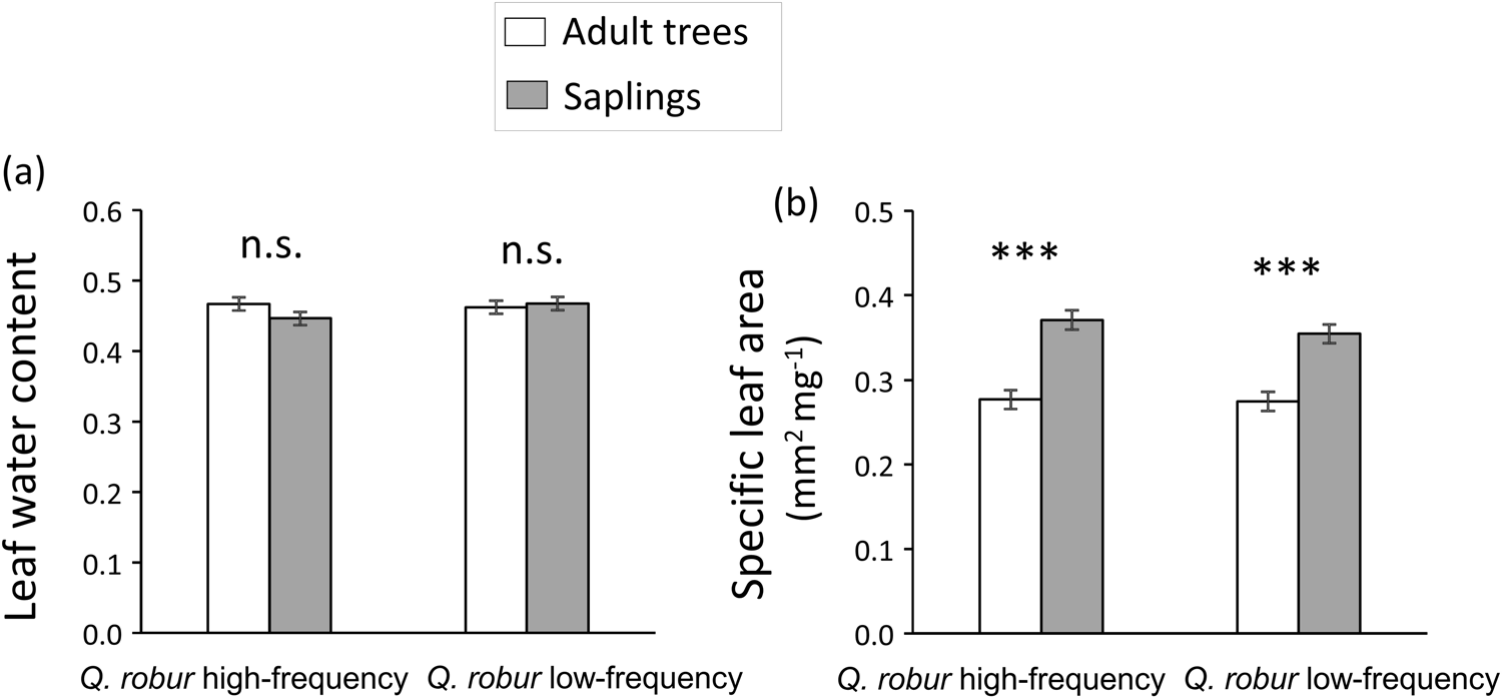
Effect of plant neighbourhood composition and ontogeny on plant physical traits in ﻿*Quercus robur﻿*. (**a**) Proportion of leaf water content and (**b**) specific leaf area (mm^2^ mg^-1^) in adult trees (white bars) and saplings (grey bars) growing in *Q. robur* high-frequency and low-frequency stands. Bars are least square means ± standard error (N = 60). Asterisks indicate significant ontogenetic differences within each *Q. robur* stand type at *P* < 0.001 (***). n.s. = non-significant.

Neighbourhood type did not significantly affect the concentration of leaf flavonoids (Table 1, Fig. 1b), lignins (Table 1, Fig. 1c), condensed tannins (Table 1, Fig. 1d), or hydrolysable tannins (Table 1, Fig. 1e), and similarly did not influence total leaf phenolics (Table 1, Fig. 1f). In contrast, we found that plant ontogenetic stage significantly affected the concentration of leaf lignins, condensed tannins, hydrolysable tannins, and total phenolics (Table 1). Specifically, the concentrations of leaf lignins, hydrolysable tannins, and total phenolics were 94%, 33% and 24% higher, respectively in saplings than in adult trees (Fig. 1c,e,f), whereas the concentration of leaf condensed tannins was instead 56% lower for saplings than adult trees (Fig. 1d); there was no significant effect of ontogeny on leaf flavonoids (Table 1, Fig. 2b). In addition, we found a significant neighbourhood type × ontogeny interaction for condensed tannins (Table 1), where the difference between adult trees and saplings was greater in high- relative to low-frequency stands (Fig. 1d). The interaction was not significant for flavonoids, lignins, hydrolysable tannins, or total phenolics (Table 1; Fig. 1b,c,e).

#### Physical traits

Neither stand type, ontogenetic stage, or their interaction significantly affected the proportion of leaf water content (Table 1, Fig. 2a). Ontogenetic stage (but not stand type or the interaction) significantly affected SLA (Table 1), where saplings exhibited a 32% greater mean value than adult trees (Fig. 2b).

### Plant traits associated with effects of neighbourhood type and ontogeny on herbivory

The effects of neighbourhood type, ontogeny, and their interaction on herbivory remained significant after including physical traits and chemical defences as covariates in the statistical model (Table 2). This suggests that neighbourhood and ontogenetic effects on herbivory were not mediated by the studied traits. Subsequently, we ran subsidiary mechanistic models including each group of phenolic compounds one at a time as covariate to uncover patterns that could be masked by using total phenolics. Results from these models indicated that the effect of ontogeny turned non-significant in the model where lignins were included (ontogeny effect: F_1,191_ = 3.86, *P* = 0.051; lignin effect: estimator: −0.0034 ± 0.0022, F_1,191_ = 2.37, *P* = 0.125); in all other cases ontogeny (and neighbourhood)  effects remained unchanged. This suggests that the difference in leaf herbivory between adult trees and saplings was mediated, to some extent at least, by the concentration of lignins in leaves. We also note that the fact that this group of phenolic compounds was negatively associated with herbivory (r = -0.18, *﻿p﻿* = 0.005, N = 240) suggests that lignins drive a reduction in herbivory and thus confer resistance in *Q. robur*.

**Table 2:** Summary of results from mixed models testing for the effects of neighbourhood type (high- vs low-frequency of *Quercus robur*), ontogenetic stage (adult trees vs. saplings), their interaction, and the effects of leaf chemical defences (total phenolics, mg g^−1^ d.w.) and leaf physical traits (proportion of water content and specific leaf area [SLA]) on insect leaf herbivory in *Q. robur*.

	Df_num,den_	F	*P*-value
Frequency	1,19	21.5	<**0.001**
Ontogeny	1,189	5.59	**0.019**
Frequency × ontogeny	1,189	5.02	**0.026**
Total phenolics	1,189	15.8	<**0.001**
Water content	1,189	1.11	0.293
SLA	1,189	0.56	0.455

Herbivory was estimated as the proportion of leaves damaged by insect herbivores for two randomly chosen low-hanging branches. These data were logit-transformed to achieve normality of residuals. Site and site × neighbourhood type were included as random factors. F-values, degrees of freedom and associated *P*-values of fixed factors are reported. Significant *P*-values (*P* < 0.05) are typed in bold.

## Discussion

Our simultaneous consideration of plant neighbourhood and ontogenetic effects on insect herbivory and plant defensive traits provides a novel contribution to research on plant-herbivore interactions. We demonstrate context-dependency of ontogenetic effects on plant defences and herbivory based on plant neighbourhood features, and also that neighbourhood effects are contingent upon a plant’s phenotype, in this case controlled by ontogeny. Specifically, we first show that leaf herbivory on *Q. robur* was lower in stands where the frequency of this focal species was low, and that saplings suffered less herbivory than adult trees. Nonetheless, we also found evidence that these two factors exerted interactive effects on herbivory. The ontogenetic effect on leaf herbivory was contingent upon neighbourhood type where the difference in damage between saplings and adult trees was only present in *Q. robur* high-frequency stands, and where the magnitude of reduction in herbivory in low- vs. high-frequency stands was greater for adults than saplings. Second, we found strong ontogenetic differences in leaf traits where saplings had thinner but more highly defended leaves than adult trees. In contrast, neighbourhood type did not influence *Q. robur* leaf traits. Third, results suggest that ontogenetic differences in leaf herbivory were mediated by leaf chemical defences such that saplings had higher concentrations of lignins and in turn received less herbivore damage. In contrast, neither neighbourhood effects on herbivory nor variation in ontogenetic effects between neighbourhood types appeared to be associated with changes in the measured plant traits.

Research conducted over the last two decades has shown that the presence of conspecific vs. heterospecific plant neighbours has strong effects on herbivore abundance and damage on focal plants^3, 5^. In support of these findings, we observed substantially lower levels of leaf herbivory on individual plants of *Q. robur* in stands with low relative frequency of this species relative to stands where this species was found at a high frequency. Several mechanisms could explain this pattern. First, a lower frequency of *Q. robur* could have driven a decrease in encounter rates between herbivores and this plant, assuming some degree of herbivore dietary specificity to this oak species (i.e., Resource Concentration Hypothesis^7, 22^). Second, higher frequencies of heterospecific neighbours in *Q. robur* low-frequency stands could have resulted in associational resistance^8, 17^ whereby one or both of the non-focal species were preferred hosts over *Q. robur* and therefore diverted herbivores away from the oak species (assuming they share some generalist herbivores) or interfered with herbivore location of *Q. robur*. Third, it is also possible that interactions with heterospecific neighbours present in higher abundances in low-frequency stands drove shifts in *Q. robur* defensive or nutritional traits (e.g., via competition or facilitation^15^) which in turn led to differences in herbivory. Our results suggest that the first two mechanisms are likely explanations for the observed pattern of herbivory, whereas the third mechanism is less probable since there were no differences in leaf physical or chemical traits between neighborhood types and the neighbourhood effect on herbivory was unaffected by the inclusion of plant traits as covariates in the herbivory statistical model. We do note, however, that our ability to test for neighbourhood control over leaf traits (particularly phenolics) may have been constrained owing to the fact that we collected leaf samples late in the growing season. For example, phenolic compounds are photo-inducible and *Q. robur* is the only deciduous species of the three found in the studied stands, such that differences in light availability between low- and high-frequency stands could be influenced by this oak’s leafing phenology. In this case, light availability (and thus phenolic production) may have been greater in high- relative to low-frequency stands in the early summer when expanding leaves of *Q. robur* are small and canopy foliage is sparse, whereas differences in light conditions between stand types would be smaller (or absent) later in the season once *Q. robur* crowns are filled with fully expanded leaves. A further characterization of the light environment between stand types early vs. late in the season and its association with leaf defensive chemistry is necessary to assess this possibility.

Our finding that adult *Q. robur* trees exhibited higher leaf herbivory than saplings is in line with previous work showing that larger and more conspicuous plants (adults in this case) are easier to find and thus suffer more damage by herbivores^23, 24^. In addition, our findings indicated that saplings were more defended than adult trees, which also agrees with theoretical expectations of increased investment during the sapling and juvenile stages, and a subsequent reduction of defences in adults^20^ (but see Barton & Koricheva^19^). The negative association between herbivory and lignins further suggests that these compounds cause a reduction in leaf damage and thus confer resistance against insect herbivores, rather than variation in herbivory driving ontogenetic differences in defences (e.g., through induction).

Interestingly, we found interactive (i.e., non-additive) effects of plant ontogeny and neighbourhood type on herbivory, where a difference in damage between adult trees and saplings was observed in stands where *Q. robur* frequency was high but not in those where this species was growing in low frequencies. We argue that the proposed difference in detectability by insect herbivores of saplings relative to adult plants in *Q. robur* high-frequency stands is superseded by a decrease in *Q. robur* frequency in low-frequency stands, which makes plants of this species overall less detectable regardless of ontogenetic stage. Only when *Q. robur* is present in a high enough frequency does ontogeny matter. Therefore, presumably both apparency and resource concentration effects presumably come into play in explaining the observed patterns of herbivory: apparency drives ontogenetic differences in damage in high-frequency stands (adults being more conspicuous), whereas resource concentration explains reduced damage in low-frequency stands as well as the contingency of ontogenetic effects on neighbourhood type (i.e., plants are consistently less attacked regardless of age when host plant frequency is low). Likewise, the magnitude of reduction in damage in low- vs. high-frequency neighbourhoods was greater for adults, suggesting that adult individuals benefit more from reductions in frequency probably because they are more conspicuous than saplings in *Q. robur* high-frequency stands.

Our findings suggest that the interactive effects of neighbourhood type and ontogeny on herbivory were not driven by changes in phenolic compounds, water content or SLA, as the interaction effect on herbivory remained significant after including plant traits in the statistical model. This suggests that the difference in leaf damage between saplings and adults in *Q. robur* high-frequency stands was not mediated by leaf traits (but rather by differences in apparency as explained above). Condensed tannins were the only leaf traits that were influenced by the interactive effects of neighbourhood type and ontogeny, but these compounds were not significantly associated with herbivory (r = 0.08, *﻿﻿P* = 0.206, N = 240). Other compounds that were significantly associated with herbivory (lignins: r = -0.18, *P* = 0.005, N = 240; hydrolysable tannins: r = -0.16, *P* = 0.014, N = 240) did not exhibit patterns that were concomitant (and could have thus been associated) with the change in ontogenetic effects across neighbourhood types. We must note, however, that further studies are needed to determine whether other unmeasured defensive traits (e.g., terpenes) in *Q. robur* were associated with (and potentially mediated) the interactive effects of neighbourhood type and ontogeny on herbivory in this oak species. It is also important to note that our measurement of herbivory represents the frequency of attack of leaves, not the actual amount of leaf area removed. Although careful field observations indicated relatively low leaf-to-leaf variation in the percent leaf area removed within as well as among individual plants (within: CV = 0.69; among: CV = 0.28), measurements of the proportion of damaged leaves may not necessarily match estimates of the amount of leaf area consumed. Therefore, these results should be interpreted exclusively in terms of frequency of leaf damage^25^.

Although there is good evidence for plant neighbourhood and plant ontogenetic effects on plant-herbivore interactions^18, 20^, few studies have looked at these effects simultaneously. Therefore, a key finding from this study is the realization that individual-level traits (phenotypic variation associated with ontogeny) and community-level properties (neighbourhood features) can interactively shape herbivory. This is to be expected since age-structured plant populations are embedded in communities with heterogeneous plant neighbourhoods that shape levels of herbivory, whereas neighbourhood effects are in turn contingent upon intra- and inter-specific phenotypic variation of focal plants. Based on this, we call for future work involving factorial experiments that test for non-additive effects arising from the joint influences of neighbourhood-level attributes and individual-level ontogenetic variation on herbivory. An important step to achieve a predictive understanding of these dynamics will be to disentangle the mechanisms producing the observed patterns, including apparency and resource concentration effects, as well as determine the causal links between plant traits and herbivory.

## Methods

### Study system

The English oak *Q. robur* is a long-lived, deciduous tree native to most of Europe^26^. At our field site (Galicia, northwestern Spain), leaf burst usually occurs in early April and leaves usually turn brown and drop off in late October. Leaves are approximately 8–12 cm long with four to seven pairs of lobes, and have a short petiole. At our field site, *Q. robur* is fed upon several insect herbivores, mainly leaf chewers such as *Tortrix viridana* (Lepidoptera: Tortricidae), *Lymantria dispar* (Lepidoptera: Lymantridae), and *Malacosoma neustria* (Lepidoptera: Lasiocampidae)^27^. Leaf miners and gall formers are less common (<5% of the leaves; X. Moreira, personal observation).

### Field sampling and leaf herbivory measurements

Towards the end of the growing season, from late September to mid October 2016, we surveyed 20 field sites in northwestern Spain which exhibited similar climatic conditions. Adjacent sites were separated by at least 10 km, and within each site we selected two stands containing at least 20 adult individuals of *Q. robur*; distance between stands within each site ranged from 1 to 3 km. At each site, one stand consisted of >85% of the adult trees represented by *Q. robur* (“*Q. robur* high-frequency stands” hereafter) whereas in the other stand <35% of the adult trees were *Q. robur* individuals (“*Q. robur* low-frequency stands” hereafter). In all stands, *Q. robur* was found growing with *Pinus pinaster* (Pinaceae) and *Eucalyptus globulus* (Myrtaceae) which were present in roughly similar relative frequencies in *Q. robur* low-frequency stands (0.346 ± 0.015 for *Eucalyptus* vs. 0.316 ± 0.015 for *Pinus*; F_1,13_ = 1.83, *P* = 0.199) and in *Q. robur* high-frequency stands (0.075 ± 0.022 for *Eucalyptus* vs. 0.057 ± 0.022 for *Pinus*; F_1,13_ = 0.32, *P* = 0.582). Although the effect of neighbourhood type was not experimentally manipulated while controlling for other unaccounted factors which could have co-varied with *Q. robur* frequency, we deliberately chose pairs of stands that were as consistent as possible in key features such as area (8287 ± 702 m^2^ for *Q. robur* high-frequency stands vs. 7431 ± 484 m^2^ for *Q. robur* low-frequency stands; F_1,38_ = 1.01, *P* = 0.322), adult tree density (202.7 ± 14.5 individuals/ha for *Q. robur* high-frequency stands vs. 230.0 ± 14.5 individuals/ha for *Q. robur* low-frequency stands; F_1,14_ = 1.77, *P* = 0.204) and sapling density (588.2 ± 73.4 individuals/ha for *Q. robur* high-frequency stands vs. 708.7 ± 73.4 individuals/ha for *Q. robur* low-frequency stands; F_1,14_ = 1.35, *P* = 0.265). In addition, we sampled all sites towards the end of the growing season to minimize phenological differences in herbivory and plant defensive traits among sites. Sampling at the end of the season also provides an assessment of cumulative leaf damage occurring over the entire growing season, considering that *Q. robur* leaf longevity spans most of the growing season^27^, and is when leaf herbivory and chemistry are more stable throughout the year^28^.

Within each stand, we randomly selected three adult trees and three saplings. Mean diameter at breast height for adults was 32.12 ± 3.67 (±SE) cm (31.68 ± 1.82 cm for *Q. robur* high-frequency stands vs. 33.78 ± 2.57 cm for *Q. robur* low-frequency stands; F_1,14_ = 0.66, *P* = 0.429) and mean diameter at root collar for saplings was 2.58 ± 0.30 cm (2.73 ± 0.22 cm for *Q. robur* high-frequency stands vs. 2.47 ± 0.22 cm for *Q. robur* low-frequency stands; F_1,14_ = 0.73, *P* = 0.407). To avoid confounding ontogeny and reproductive status (i.e., variation between reproductive vs. non-reproductive adults), we selected adult trees with no (or few) acorns produced during the current year. Distance among individuals within stands was at least 10 m. In total, we sampled 240 trees corresponding to 20 sites × two *Q. robur* neighbourhood types × two ontogenetic stages × three individual trees.

For each adult tree, we visually inspected leaf herbivory for two randomly chosen low-hanging branches (1 to 2 m above ground level). We estimated the proportion of herbivore-damaged leaves by randomly choosing 25 leaves per branch and counting the number of leaves attacked by insect herbivores^25, 27^. Previous observations at each site indicated that the proportion of herbivore-damaged leaves was evenly distributed throughout the canopy (0.296 ± 0.088 for low-hanging branches vs. 0.336 ± 0.088 for the rest of the canopy; F_1,17_ = 0.22, *P* = 0.644) and therefore sampling low-hanging branches was a good proxy of damage at the whole-tree level^27^. In the case of saplings, we visually counted the number of herbivore-damaged leaves throughout the entire canopy and calculated the proportion of damaged leaves for the whole plant. In all cases leaf herbivory damage was caused by insect herbivores. Mammalian herbivores are not very common in the studied region, and we did not find signs of leaf herbivory by mammals in either saplings or adult trees.

After recording leaf herbivory, we collected three fully expanded leaves per branch for adult individuals, and six fully expanded leaves in the terminal leader for saplings to quantify leaf chemical and physical traits (see ahead). In all cases, we selected leaves with little or no evidence of insect damage or pathogen infection to reduce variation in defences caused by site-specific induction. However, sampling undamaged leaves does not eliminate systemic induced responses. Therefore, the concentration of chemical defences measured likely represented a mixture between constitutive levels of defence plus some unknown level of induction due to systemic responses.

The above sampling scheme followed a randomized split-plot design replicated across 20 sites, with *Q. robur* frequency stand (two levels: high and low frequency) as the whole factor and plant ontogenetic stage (two levels: adult trees and saplings) as the split factor.

### Quantification of leaf physical traits and chemical defences

#### Physical traits

Immediately after leaf collection, we weighted fresh leaves and oven-dried the samples for 48 h at 40 °C until constant weight was achieved. We then weighted the dry leaves and estimated the proportion of leaf water content ([dry weight/fresh weight]) of each plant. We also calculated specific leaf area (SLA) for each plant by dividing the surface area of a 9.5-mm diameter disk by its dry mass in mg. We only measured a single leaf per plant because previous trials demonstrated relatively low leaf-to-leaf variation within individual plants. In particular, we found that the coefficient of variation within-individual trees ranged from moderate to low for both variables (SLA: CV = 0.73, water content: CV = 0.62). Water content (physiologically limiting for herbivores) and SLA (correlated with toughness and thus palatability) are both associated with leaf quality to insect herbivores. Previous work in other systems has shown that low values for both variables was associated with decreased leaf nutritional quality and palatability for insect herbivores^29^.

#### Chemical defences

After measuring physical traits, we grinded the leaves with liquid nitrogen for quantification of phenolic compounds. We chose phenolic compounds because previous work has reported that they confer resistance against insect herbivores in *Q. robur* ^30, 31^. We extracted phenolic compounds using 20 mg of dry plant tissue with 0.8 mL of 70% methanol in an ultrasonic bath for 15 min, followed by centrifugation^32^. We diluted these methanolic extracts (1:6 vol:vol) with the extraction solvent and transferred them to chromatographic vials. We performed phenolic profiling according to Moreira *et al*.^33^. Briefly, we used ultrahigh-pressure liquid chromatography-quadrupole-time-of-flight mass spectrometry (UHPLC-QTOF-MS) to detect, identify and quantify phenolic compounds. The separation was carried out on a 50 × 2.1 mm Acquity UPLC BEH C18 column (Waters, Milford, CT, USA) using a gradient composed of water +0.05% vol. formic acid (solvent A) and acetonitrile +0.05% vol. formic acid (solvent B). The QTOF-MS (Synapt G2, Waters) was operated in MS^E^ negative mode over an m/z range of 85–1200 Da and internally calibrated by infusing a solution of leucine-enkephaline through the Lock Spray^TM^ probe. We identified phenolic compounds on the basis of their molecular formula (as determined from exact mass measurements), fragment ions, and comparison with available databases such as the Dictionary of Natural Products (Chapman & Hall, CRC Informa, London; version 20.2) or ReSpect for Phytochemicals. We identified four groups of phenolic compounds (flavonoids, condensed and hydrolysable tannins and lignins). We quantified flavonoids as rutin equivalents, condensed tannins as catechin equivalents, hydrolysable tannins as gallic acid equivalents, and lignins as ferulic acid equivalents. We achieved the quantification of these phenolic compounds by external calibration using calibration curves at 0.2, 0.8, 2, 5 and 20 μg/mL. We calculated total phenolics as the sum of flavonoids, lignins, condensed tannins and hydrolysable tannins, and expressed phenolic compound concentrations in mg g^−1^ tissue on a dry weight basis.

### Statistical analyses

#### Effects of neighbourhood type and ontogeny on Q. robur herbivory and leaf traits

We analysed the effects of neighbourhood type, plant ontogeny, and their interaction on leaf herbivory (proportion of damaged leaves), leaf physical traits (proportion of leaf water content and SLA), and leaf chemical defences (total phenolics and separately for each group of phenolic compounds) with the proper mixed models solving for a split-plot design using PROC MIXED in SAS (SAS 9.4, SAS Institute, Cary, NC)^34^. Neighbourhood type (*Q. robur* high- vs. low frequency), ontogenetic stage (*Q. robur* adult trees vs. saplings), and their interaction were considered fixed factors. Site and site × neighbourhood type were included as random factors where the latter interaction specifies the appropriate test of neighbourhood type effect using stand as unit of replication (i.e., whole plot factor)^34^. We logit-transformed herbivory and water content data to achieve normality of residuals; in all other cases, residuals were normally distributed.

#### Plant traits associated with effects of neighbourhood type and ontogeny on herbivory

To determine whether the measured leaf traits were associated with effects of neighbourhood type and *Q. robur* ontogeny on leaf damage, we ran again the same herbivory model described above but now including as covariates total phenolics, water content, and SLA. We chose to include these three traits because they are weakly correlated (r = 0.08 to 0.23), therefore reducing the influence of colinearity. We expected that if physical traits or chemical defences mediate effects of neighbourhood type and ontogenetic stage on leaf herbivory, then significant effects of any of these factors (or their interaction) should turn non-significant once such traits are accounted for in the model. If neighbourhood type and ontogenetic stage effects remain significant after including these traits, this suggests that these factors influence herbivory through other unmeasured plant traits or via some other mechanism not associated with plant trait variation. Subsequently, we ran subsidiary mechanistic models including each group of phenolic compounds at a time as covariate (total of four models, one per covariate) to uncover patterns that could be masked by using total phenolics. We used this approach instead of simultaneously including all groups of phenolics in a single model, because some of these compounds are highly correlated and this may complicate separating and testing for their individual effects on herbivory.
